# P-1002. Congenital Syphilis: Are We Doing Too Much?

**DOI:** 10.1093/ofid/ofae631.1192

**Published:** 2025-01-29

**Authors:** Haley Anne Palaganas, Alexandra Iacob, Jagmohan S Batra, Ching Ching Tay, Antoine Soliman, Leonel F Guajardo

**Affiliations:** MemorialCare, Orange, California; MemorialCare Miller Children's & Women's Hospital Long Beach, Long Beach, California; University of California, Irvine School of Medicine, Long Beach, California; Me, Cypress, California; "Miller Children's and Women's Hospital, Long Beach, California; Miller children’s hospital Long Beach CA, Irvine, California

## Abstract

**Background:**

The resurgence of syphilis and its risks to neonates has been reported nationally. Sixty to ninety percent of infected infants can be asymptomatic at birth. Effective early identification and treatment of congenital syphilis is paramount. The American Academy of Pediatrics (AAP) offers guidelines for categorizing risk of congenital syphilis and outlines recommended and optional testing for completed workup. Literature on adherence to these guidelines is limited. We hypothesized that unnecessary imaging and labs were obtained on infants without an impact in their management.

**Figure:**
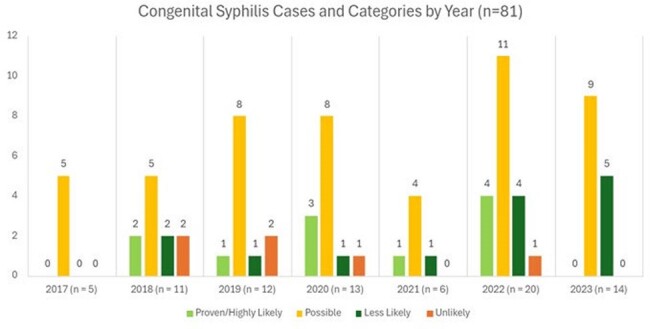

Number of congenital syphilis cases per year and divided by diagnostic category.

**Methods:**

We conducted a retrospective study of all infants with a diagnosis of congenital syphilis admitted at MemorialCare Miller Children’s and Women’s Hospital, an urban tertiary referral center, from Jan 2017 – Dec 2023 (IRB # MHS 361-23). Infants were subdivided by congenital syphilis diagnostic category. Data collected for infants included completion of long bone X-ray, head ultrasound, MRI brain, ophthalmologic exams, and lumbar puncture with CSF analysis, as well as if management changes were made based off study results and if any of the studies were abnormal.

**Figure:**
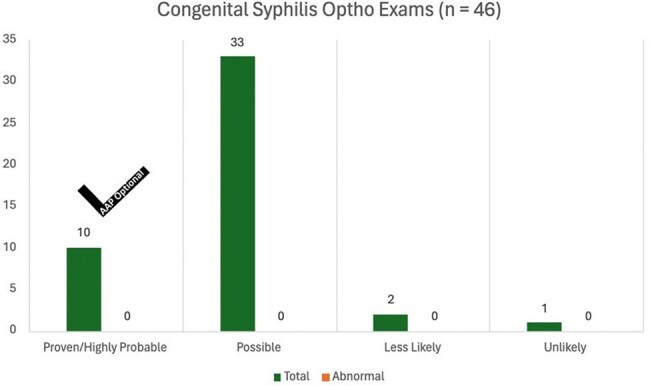

Number of ophthalmology exams ordered for each congenital syphilis diagnostic category. The black checkmark indicates if this study was recommended by the AAP.

**Results:**

81 infants were identified in this period. 11 (13.6%) had proven or highly likely, 50 (61.7%) had possible, 14 (17.3%) had likely, and 6 (7.4%) had less likely congenital syphilis. 46 (56.8%) received ophthalmologic exams, all of which did not lead to changes in management. 36 of the 46 ophthalmologic exams (78.3%) were conducted without recommendations from the AAP. 10 out of 19 (52.6%) brain MRIs were ordered without AAP recommendations and did not result in changes in management. 4 out of 62 (7%) long bone x-rays were performed without either AAP recommendation or subsequent changes in management. Similarly, 5 out of 66 (7.6%) lumbar punctures were carried out without adherence to AAP recommendations and did not result in changes in management. 12 out of 15 (80%) head ultrasounds were conducted without AAP recommendation.

**Figure:**
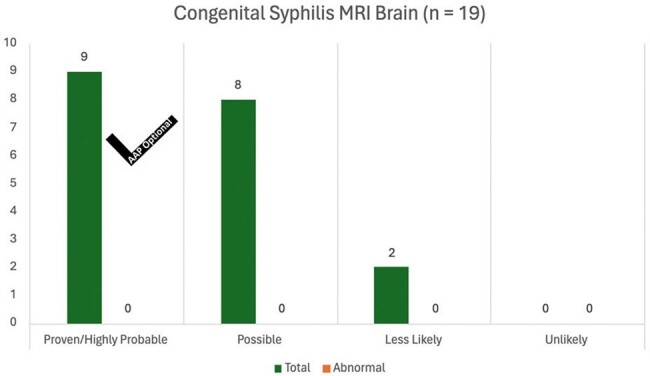

Number of brain MRIs ordered for each congenital syphilis diagnostic category. The black checkmark indicates if this study was recommended by the AAP.

**Conclusion:**

Unnecessary labs and imaging were frequently ordered for congenital syphilis work up. Our unit has now undertaken a multi-disciplinary quality improvement project to ensure timely and proper categorization of congenital syphilis risk to ensure appropriate imaging and labs are obtained.

**Disclosures:**

**All Authors**: No reported disclosures

